# Non-linear association of sleep duration with osteoarthritis among middle-aged and older adults: Results from a prospective cohort study in China

**DOI:** 10.1371/journal.pone.0335552

**Published:** 2025-10-31

**Authors:** Long Long, Qi Wang, Bohao Fang, Li Cao, Yingjie Xu, Guangyang Li

**Affiliations:** Department of Orthopedic, Linping Hospital of Integrated Traditional Chinese and Western Medicine, Hangzhou, Zhejiang, People's Republic of China; The First Affiliated Hospital of Soochow University, CHINA

## Abstract

**Background:**

The association between nighttime sleep duration and osteoarthritis (OA) remains ambiguous. Chinese older adults exhibit distinct sleep patterns, as well as genetic predispositions and rapidly changing lifestyles, which may have shaped the unique epidemiology of OA. However, most existing evidence is based on Western populations. This study aimed to investigate this association and the potential moderating role of BMI in middle-aged and older Chinese adults.

**Methods:**

Data of wave 1 (2011) and wave 4 (2018) were obtained from the nationally representative China Health and Retirement Longitudinal Study (CHARLS). Univariate and multivariate logistic regression models were utilized to explore the association of sleep duration and OA with sleeping 7–9 h as reference group. Additionally, to further explore the potential combined effect of sleep and BMI, interaction terms were added into the model. Restricted cubic spline was also used to explore the non-linear correlation between sleep duration and OA.

**Results:**

Out of 6,825 participants, 1,396 were diagnosed with OA. After multivariable adjustment, the odds ratios (OR) for OA were 1.39 (95% CI 1.20–1.60; P < 0.001) for individuals with sleep duration (<6 h/night) and 1.27 (95% CI 1.20–1.60 P = 0.003) for individuals with sleep duration (6–7 h/night). The association between sleep duration and OA followed a U-shaped pattern, with 7.5 h acting as an inflection point. Significant interactions were found in overweight individuals, with both short (OR = 1.41, P = 0.042) and long (OR = 2.71, P = 0.006) sleep durations increasing OA risk.

**Conclusions:**

Short sleep duration (<7h) was associated with a higher incidence of OA. A U-shaped association was observed between sleep duration and OA incidence among middle-aged and older Chinese adults. BMI may act as a moderator in this relationship.

## Introduction

Osteoarthritis (OA), a prevalent degenerative joint disease characterized by articular cartilage deterioration, pain, and functional impairment, poses a significant global health burden. OA affects over 303 million individuals worldwide and disproportionately impacts middle-aged and older adults. Its prevalence is expected to rise due to aging populations and increasing obesity rates [1]. In China, rapid urbanization, significant lifestyle transitions, and an increasing obesity rate have notably increased the burden and altered the risk factor profile for OA compared to previous decades, highlighting the need for targeted epidemiological insights [2]. Additionally, Chinese older adults exhibit unique sleep patterns, including a higher prevalence of daytime napping compared to Western populations (approximately 60% vs. 25%, respectively) [3]. Furthermore, genetic and epigenetic differences in OA susceptibility among Asian populations compared to Europeans underscore the necessity for region-specific studies [4]. Despite these distinctive characteristics, most existing epidemiological studies investigating sleep duration and OA primarily focus on Western cohorts, limiting generalizability to the Chinese population. Beyond its economic costs, OA significantly reduces quality of life through chronic pain and mobility limitations, necessitating deeper insights into modifiable risk factors [3].

Sleep, a cornerstone of physiological homeostasis, plays a pivotal role in systemic health. Current guidelines recommend 7–9 hours of nightly sleep for adults, yet nearly one-third of individuals report suboptimal sleep duration [5]. Both short (<7 hours) and long (>9 hours) sleep durations have been linked to adverse outcomes, including cardiovascular diseases, metabolic dysfunction, and premature mortality [6]. Emerging evidence suggests sleep disturbances may also influence OA pathogenesis. Mechanistically, disrupted sleep elevates pro-inflammatory cytokines such as interleukin-6 (IL-6) and tumor necrosis factor-alpha (TNF-α), which accelerate cartilage degradation and synovial inflammation [7]. Epidemiological studies reveal a U-shaped association between sleep duration and OA risk, with minimal risk observed at 7–8 hours [8,9]. However, these findings derive predominantly from Western cohorts, limiting generalizability to Asian populations with distinct lifestyle and genetic profiles [4].

Notably, obesity—quantified by body mass index (BMI), is a key driver of systemic inflammation and metabolic dysregulation [10]. Visceral fat secretes adipocytokines (e.g., leptin, adiponectin) that promote joint catabolism, while excess mechanical load on weight-bearing joints exacerbates structural damage [10]. Both insufficient and prolonged sleep correlate with elevated BMI, suggesting bidirectional pathways linking sleep, adiposity, and OA [11,12]. Previous studies in U.S. cohorts have shown that 12.1% of the association between sleep duration and OA is mediated by waist circumference [13]. However, a study on nighttime sleep duration and knee OA in a Chinese population lacked in-depth analysis of the moderating role of BMI [2].

To address these gaps, we leveraged data from a prospective cohort of middle-aged and older adults in China to investigate: (1) the relationship between sleep duration and OA incidence, and (2) the moderating role of BMI.

## Method

### Study population

We utilized data from the CHARLS [14], a nationally representative longitudinal study of Chinese adults aged 45 and above. The baseline national census of CHARLS was fielded in 2011, including about 10,000 households from 17,500 individuals in 150 counties/districts and 450 villages/resident committees through multistage stratified probability-proportionate-to-size sampling. The individuals were followed up every 2 or 3 years by a face-to-face computer-assisted personal interview. More details about objectives, design, and methods can be found in a previous study [14].

**Fig 1:**
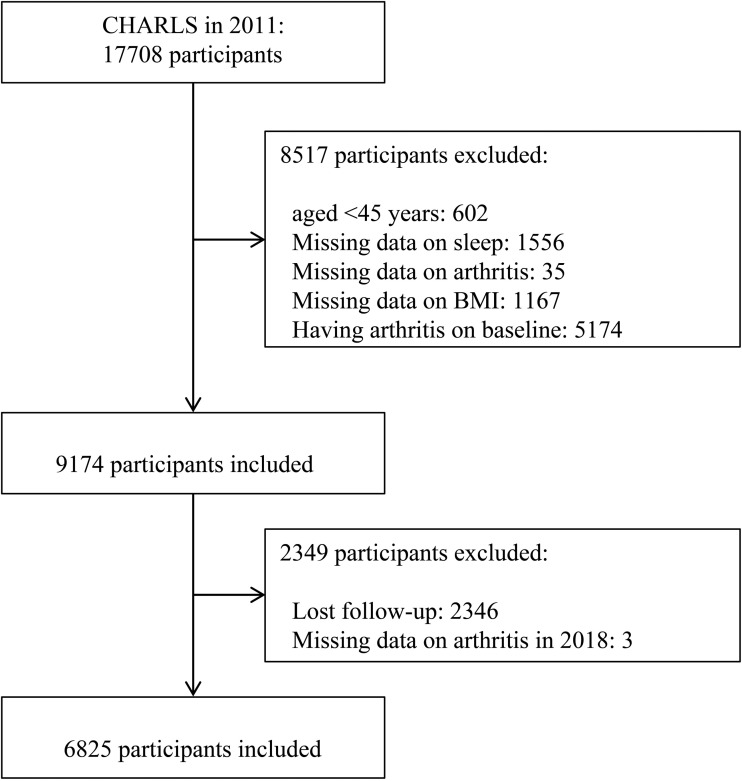
Flowchart of participant selection in the present study. Abbreviations: CHARLS, China Health and Retirement Longitudinal Study; BMI, body mass index.

The inclusion criteria were: 1) individuals aged at least 45 years old in the baseline survey; 2) having data regarding OA, nighttime sleep duration, and BMI; 3) reported having OA absence in the baseline; 4) being followed up till 2018; 5) and having OA data in 2018. After exclusion, 6,825 respondents were included in the analysis. The detailed process of participant selection is shown in Fig 1.

The CHARLS had received ethical approval from the Institutional Review Board of Peking University (approval number: IRB00001052-11,015) [14] Written informed consents were obtained from all participants before attending the survey. The CHARLS was performed in line with the Declaration of Helsinki. This study was conducted following the Strengthening the Reporting of Observational Studies in Epidemiology guideline.

### Assessment of sleep duration

The data on nighttime sleep duration was collected in the 2011 baseline survey. Each participant was asked “During the past month, how many hours of actual sleep did you get at night (average hours for one night)?”. We further categorized the self-reported nighttime sleep duration into four groups: < 6 h, 6 to < 7 h, 7–9 h, and > 9 h[5]. Sleep duration of 7–9 h was treated as the reference group.

### Assessment of arthritis

New-onset arthritis was identified based on self-reported data; when the interviewer asked, “Have you been diagnosed with arthritis by a doctor?” and the respondents answered “Yes,” they were classified as arthritis patients. Participants who had arthritis in 2011 were excluded, and if the patient was diagnosed with arthritis after that until the follow-up period in 2018, he or she was included in the study under our definition of a patient with new-onset arthritis.

### Assessment of covariates

The process of selecting covariates is grounded in substantial reasoning and prior scholarly works [15–17]. Covariates included age, gender, current marriage status (married or single), education level (no formal education, elementary school or lower, middle school, and high school or above), household income per capita (log transformed), smoking status, drinking status were collected through face-to-face interviews. BMI was categorized according to the World Health Organization (WHO) classification [18]: underweight (<18.5 kg/m^2^), normal weight (between 18.5 and less than 25 kg/m^2^), overweight (between 25 and less than 30 kg/m^2^), and obesity (≥30.0 kg/m^2^). Waist circumference was accessed using a soft measure tape while standing [14]. The diagnoses of hypertension and diabetes mellitus (DM) were based on self-reported medical history. The high sensitivity C-reactive protein (CRP) was measured by the immunoturbidimetric assay and the white blood cell (WBC) was measured by automated analyzers within 141 min [14].

### Statistical analysis

Continuous variables were presented as means ± standard deviations (SDs and categorical variables were described as frequencies and proportions. Differences between groups were compared by independent sample t-text for continuous variables and chi-square test for categorical variables. As this analysis compares traits across independent groups (not within subjects over time), independence assumptions for t-tests/chi-square were satisfied.

In the correlation analysis, we used the multivariate logistic regression model to estimate the odds ratios (OR) with 95% confidence interval (95% CI) for the association of nighttime sleep duration with incident OA. We used three models in the analyses, of which Model 1 was the crude model. Model 2 adjusted for age and gender. Model 3 additionally adjusted current marriage status, education level, household income per capita, smoking and drinking status, BMI, waist circumference, hypertension, DM, CRP and WBC level based on model 2.

The study also examined the non-linear association between sleep duration and OA using restricted cubic spline. The saturation value of the connection between sleep duration and OA was determined using threshold effect analysis. We also conducted subgroup analysis by dividing the participants by sex and age in order to investigate the correlation between sleep duration and OA in various subgroups. Data were analyzed using R (version 3.4.3). P < 0.05 was chosen as the level of significance for the analysis.

## Results

### Baseline characteristics of study population

After applying the inclusion and exclusion criteria, a total of 6,825 participants were included in the study. The mean age of participants without OA was 57.6 ± 8.8 years, while for those with OA, it was 58.2 ± 8.7 years. Among participants with OA, 43.8% were female, while 56.2% were male. Individuals with OA more often reported sleeping < 6 h compared to participants without OA. A summary of the baseline characteristics of the subjects is provided in Table 1.

**Table 1:** Characteristics of the study population.

	Total (N = 6825)	Arthritis		P value
		No (N = 5429)	Yes (N = 1396)	
**Age (mean, SD)**	57.7 (8.8)	57.6 (8.8)	58.2 (8.7)	0.039
**Sex**				p < 0.001
Female	3404 (49.9%)	2793 (51.4%)	611 (43.8%)	
Male	3421 (50.1%)	2636 (48.6%)	785 (56.2%)	
**Marriage**				0.083
Married	6119 (89.7%)	4885 (90%)	1234 (88.4%)	
Single	706 (10.3%)	544 (10%)	162 (11.6%)	
**Household consumption**	8.5 (0.8)	8.6 (0.8)	8.5 (0.8)	0.035
**Education**				p < 0.001
No formal education	1765 (25.9%)	1354 (24.9%)	411 (29.4%)	
Elementary school or lower	2667 (39.1%)	2061 (38%)	606 (43.4%)	
Middle school	1538 (22.5%)	1279 (23.6%)	259 (18.6%)	
High school and above	855 (12.5%)	735 (13.5%)	120 (8.6%)	
**Smoking**				0.043
Non-smoker	4023 (58.9%)	3167 (58.3%)	856 (61.3%)	
Smoker	2802 (41.1%)	2262 (41.7%)	540 (38.7%)	
**Drinking**				0.014
Non-drinker	4793 (70.2%)	3779 (69.6%)	1014 (72.6%)	
Drinking less than once a month	1249 (18.3%)	1031 (19%)	218 (15.6%)	
Drinking more than once a month	783 (11.5%)	619 (11.4%)	164 (11.7%)	
**Hypertension**				0.086
No	5294 (78.0%)	4235 (75.9%)	1194 (77.6%)	
Yes	1531 (22.0%)	1194 (24.1%)	337 (22.4%)	
**Diabetes**				0.236
No	6464 (94.7%)	5133 (94.5%)	1331 (95.3%)	
Yes	361 (5.3%)	296 (5.5%)	65 (4.7%)	
**WBC (10^9/L) (mean, SD)**	6.21 (1.57)	6.20 (1.56)		0.619
**CRP (mg/l) (mean, SD)**	2.42 (5.83)	2.42 (6.08)	2.39 (4.74)	0.867
**Sleep duration**				p < 0.001
< 6 h	1723 (25.2%)	1299 (23.9%)	424 (30.4%)	
[6, 7] h	1443 (21.1%)	1131 (20.8%)	312 (22.3%)	
7–9 h	3371 (49.4%)	2761 (50.9%)	610 (43.7%)	
> 9 h	288 (4.2%)	238 (4.4%)	50 (3.6%)	
**Obesity**				0.58
Underweight	398 (5.8%)	313 (5.8%)	85 (6.1%)	
Normal weight	4359 (63.9%)	3485 (64.2%)	874 (62.6%)	
Overweight	1745 (25.6%)	1382 (25.5%)	363 (26%)	
Obesity	323 (4.7%)	249 (4.6%)	74 (5.3%)	

Mean ± SD for continuous variables: the P value was calculated by using t-test; (%) for categorical variables: the P value was calculated by chi-square test.

Abbreviation: BMI, body mass index; WBC, White Blood Cell count; CRP, C-Reactive Protein.

### Associations between sleep duration and OA

Table 2 presents the analysis of the association between sleep duration and OA. The ORs 95% CI for OA is reported for different sleep durations, compared to appropriate sleep duration (7–9 h/night). In model 1, which remained unadjusted for any variables, the duration of sleep exhibited a statistically significant association with OA as a categorical variable. Participants who slept <6 h had a 1.48-fold higher risk of developing OA compared to those who slept 7–9 h (OR = 1.48, 95% CI: 1.28–1.70, p < 0.001). Conversely, there was no noteworthy variance in OA incidence between participants who slept for > 9 h and those who slept for 7–9 h (OR = 0.95, 95% CI [0.69–1.31] p = 0.756). In Model 3, after accounting for all covariates, participants who slept <6 h had a 1.39-fold higher risk of developing OA compared to those with appropriate sleep durations (OR = 1.39, 95% CI: 1.20–1.60, p < 0.001).

**Table 2:** Association between sleep duration and osteoarthritis in multiple regression model.

	Sleep duration			
	<6 h	[6,7] h	7–9 h	>9 h
Multiple regression model				
Model 1	1.48 (1.28–1.70)	1.25 (1.07–1.45)	1.00 (reference)	0.95 (0.69–1.31)
	P < 0.001	P = 0.004		P = 0.756
Model 2	1.43 (1.24–1.65)	1.25 (1.07–1.46)	1.00 (reference)	0.94 (0.69–1.30)
	P < 0.001	P = 0.004		P = 0.716
Model 3	1.39 (1.20–1.60)	1.27 (1.20–1.60)	1.00 (reference)	0.91 (0.66–1.25)
	P < 0.001	P = 0.003		P = 0.549

Values are odds ratios (ORs) with 95% confidence intervals (CIs). The 7–9 h sleep duration group was used as the reference category. P values are for comparisons with the reference group.

Model 1: no covariates were adjusted. Model 2: age, and sex were adjusted. Model 3: age, sex, current marriage status, education level, household income per capita, smoking and drinking status, hypertension, diabetes, white blood cell count, C-reactive protein and BMI were adjusted.

There were significant interactions among subgroups of sleeping <6 hours with overweight status (OR = 1.41, P = 0.042) and sleeping >9 hours with overweight status (OR = 2.71, P = 0.006). No significant interactions were observed in the underweight and obesity groups across different sleep durations (Table 3).

**Table 3:** The interaction effects of sleep duration and BMI.

Sleep duration and BMI	OR	P-value	95% CI	
			Lower	Upper
<6 h and underweight	1.28	0.421	0.70	2.32
<6 h and overweight	1.41	0.042	1.01	1.98
<6 h and obesity	0.70	0.327	0.34	1.43
[6,7] h and underweight	1.07	0.848	0.53	2.17
[6,7] h and overweight	1.22	0.282	0.85	1.74
[6,7] h and obesity	1.53	0.203	0.79	2.96
>9 h and underweight	2.89	0.066	0.93	8.98
>9 h and overweight	2.71	0.006	1.32	5.54

Values are odds ratios (ORs) with 95% confidence intervals (CIs). P values are derived from multivariable logistic regression models. The group with 7–9 h of sleep and normal BMI was used as the reference category. Analyses were adjusted for age, sex, current marriage status, education level, household income per capita, smoking and drinking status, hypertension, diabetes, white blood cell count, C-reactive protein and BMI.

Abbreviations: BMI, body mass index; OR, odds ratio; CI, confidence interval.

### Analysis of threshold effects and smooth curve fitting

The threshold analysis identified 7.5 h as the inflection point, with a negative association below this point (β = −0.014, p < 0.05) and a positive association above it (β = 0.032, p < 0.05).

**Fig 2:**
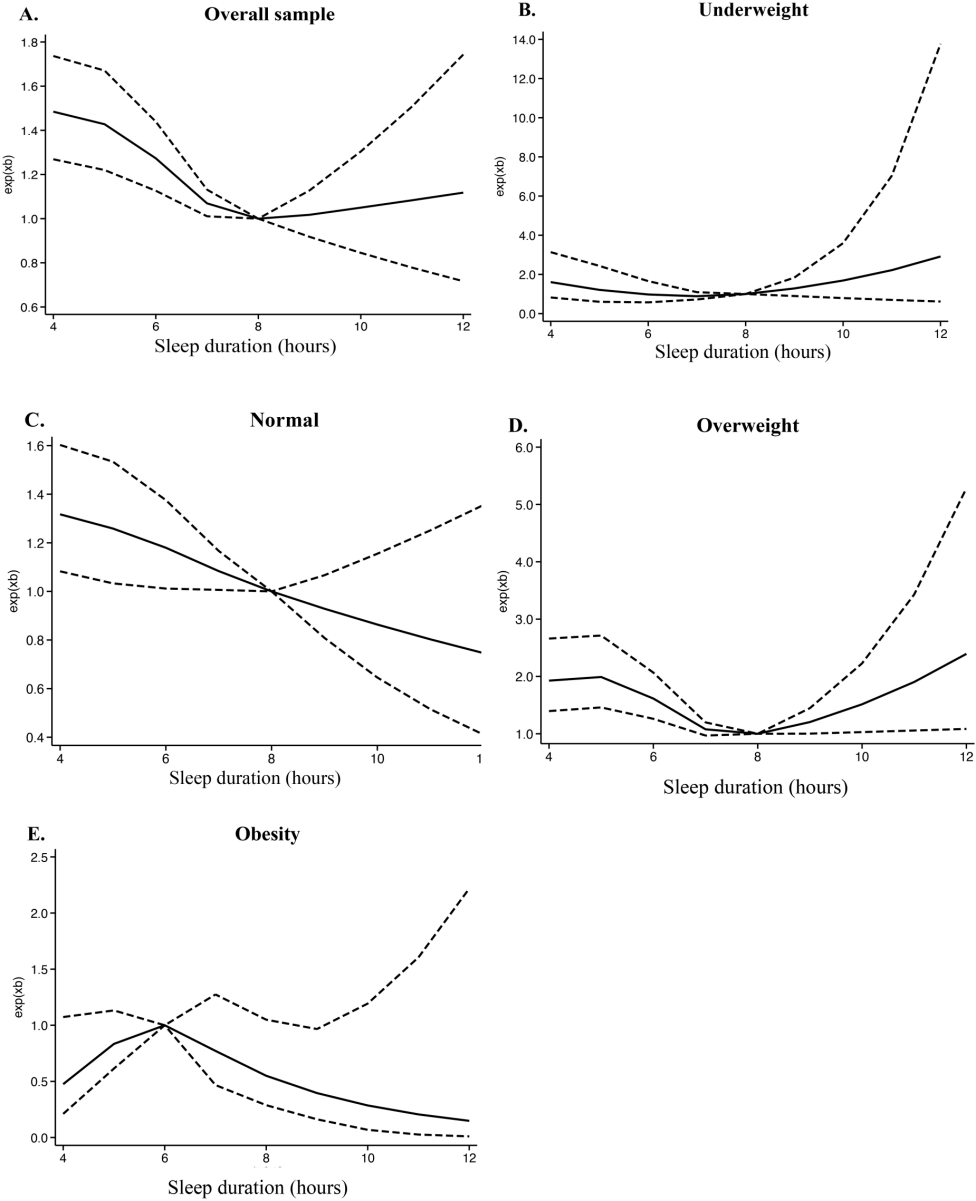
Non-linear association between sleep duration and the risk of OA in the overall and BMI-stratified populations. (A) overall sample, (B) underweight, (C) normal weight, (D) overweight, and (E) obesity. Abbreviations: OA, osteoarthritis; BMI, body mass index.

Fig 2(A) depicts a U-shaped association between the overall occurrence of OA and the duration of sleep. Non-linear correlation between sleep duration and OA in the categorized BMI groups are presented in Fig 2(B-E). Among individuals with different BMI levels, sleep duration influenced changes in OA occurrence in distinct ways. For those who were overweight, the U-shaped curve was more pronounced, whereas the obesity group exhibited an inverted U-shaped curve. In normal-weight individuals, the occurrence of OA gradually decreased as sleep duration increased. However, this variation appeared non-significant in the underweight group.

### Subgroup analysis

Subgroup analysis indicated that the association between sleep duration and OA differed by sex and age group (Table 4). In males, compared to those with a sleep duration of 7–9 hours, individuals with short sleep duration (<6 hours) had a 30% higher likelihood of developing OA (OR = 1.30, 95% CI [1.04–1.62], p = 0.019). Among females, both short and insufficient sleep duration were significantly associated with an increased risk of OA. Those sleeping less than 6 hours had a 45% higher OA risk (OR = 1.45, 95% CI [1.20–1.76], p < 0.001), while those sleeping between 6 and 7 hours had a 31% increased risk (OR = 1.31, 95% CI [1.06–1.63], p = 0.012). However, prolonged sleep duration (>9 hours) was not significantly associated with OA in both males and females. Similar results like main models were also observed in the subgroup analysis by age.

**Table 4:** Subgroup analysis of the association between sleep duration and osteoarthritis.

	Sleep duration			
	<6 h	[6,7] h	7-9 h	>9 h
**Sex**				
** Male**	1.30 (1.04–1.62)	1.20 (0.96–1.50)	1.00 (reference)	1.18 (0.77–1.82)
	P = 0.019	P = 0.108		P = 0.77
** Female**	1.45 (1.20–1.76)	1.31 (1.06–1.63)	1.00 (reference)	0.68 (0.42–1.11)
	p < 0.001	P = 0.012		P = 0.121
**Age**				
** 45**–**60**	1.39 (1.15–1.68)	1.30 (1.07–1.58)	1.00 (reference)	1.00 (0.67–1.49)
	P = 0.001	P = 0.008		P = 0.983
** ≥ 60**	1.41 (1.13–1.75)	1.24 (0.96–1.59)	1.00 (reference)	0.79 (0.47–1.35)
	P = 0.002	P = 0.100		P = 0.388

Values are odds ratios with 95% confidence intervals. The 7–9 h sleep duration group was used as the reference category. P values are for comparisons with the reference group. Analyses were adjusted for age, sex, current marriage status, education level, household income per capita, smoking and drinking status, hypertension, diabetes, white blood cell count, C-reactive protein and BMI.

## Discussion

Through this cross-sectional analysis, we found short sleep (<7h) was associated with a higher incidence of OA compared to normal night-time sleep duration (7-9h). Additionally, we further identified a non-linear, U-shaped association between sleep duration and the risk of developing OA in middle-aged and older Chinese adults, and this association exhibits significant variations across different body weight subgroups. A sleep duration of approximately 7.5 hours appears optimal for reducing the risk of OA.

Our findings demonstrate a significant association between sleep duration and OA, consistent with previous studies. Importantly, several Asian cohort studies support this relationship. For example, Cho et al. analyzed 8,918 Korean adults aged ≥50 years and found that both short (≤6 hours/day) and long (≥9 hours/day) sleep durations were associated with higher OA prevalence, although the association with long sleep lost significance after adjustment. However, subgroup analysis showed that participants with both radiological OA and joint pain had increased odds of OA with both short and long sleep durations (OR=1.32 and 1.41, respectively) [19]. In a Chinese study, Zhou et al. (2024) observed that those with <6 hours or >9 hours of sleep had higher incidence of knee OA over four years, though the trend was stronger for short sleep duration [2]. These Asian studies closely align with our findings and suggest the possibility of a U-shaped association in regional populations.

In contrast, several European studies [8,20] observed similar U-shaped trends in Western cohorts, but often based on different lifestyle baselines. For instance, Jung et al., in a sample of 11,540 participants, reported increased OA prevalence with both <6 and ≥8 hours of sleep, though the association for long sleep did not reach significance [8]. These discrepancies may reflect cultural and behavioral differences. Notably, approximately 60% of older Chinese adults take regular daytime naps, compared to about 25% in the U.S., potentially affecting nighttime sleep duration measurements [21]. Further research is warranted to clarify the impact of prolonged sleep on OA development in different populations.

Our analysis also indicates that BMI moderates the association between sleep duration and OA risk. Previous work using the NHANES database found that 12.1% of the association between sleep duration and OA was mediated by waist circumference [13]. In our study, visualization of the BMI subgroups revealed that, although a U-shaped association was evident in the overall population, the curve was more pronounced in the overweight subgroup, whereas an inverted U-shaped relationship was observed in the obese subgroup. Normal-weight individuals exhibited a gradual decrease in OA incidence with increasing sleep duration, while the underweight group showed no significant variation. The inverted U-shaped curve in obese individuals is intriguing and may be explained by several factors: (1) the potential masking of the true effect by an underlying inflammatory state, whereby individuals with moderate sleep duration may be in a “balanced” condition; and (2) the higher prevalence of sleep disorders such as sleep apnea in overweight populations [22], which may complicate the relationship between reported sleep duration and actual sleep quality. Additionally, the obesity rate among Chinese older adults (approximately 9%) is significantly lower than that in the United States (41%) and Europe (20%−30%) [23]. This may partially explain why the effect of obesity in our study was less pronounced than that reported by Ma et al. [13]. These findings underscore the importance of developing targeted OA prevention and management strategies that consider variations in body weight.

Regarding underlying mechanisms, a prevailing explanation involves the activation of pro-inflammatory pathways and oxidative stress induced by extreme sleep durations, leading to increased expression of cytokines such as interleukin-6 (IL-6) and tumor necrosis factor-α (TNF-α) [24]. These cytokines are critical mediators in the degradation of cartilage matrix and the pathogenesis of OA [25]. Moreover, the relationship between poor sleep quality and OA may be bidirectional and complex, especially in the context of metabolic disorders such as obesity, type 2 diabetes [26], and hypertension [27]. Notably, overweight and obese individuals typically exhibit elevated plasma levels of TNF-α and IL-6 [28], which, in addition to increased mechanical stress on joints [29], may further contribute to cartilage damage and OA progression. Importantly, our study included multiple OA sites (e.g., knee, spine, hand), suggesting that chronic inflammation associated with abnormal sleep duration may exert a generalized effect on OA development.

### Limitation

Several limitations of our study should be acknowledged. First, variables regarding sleep duration and OA were self-reported in CHARLS, without clinical examination or imaging confirmation, which may introduce recall bias and misclassification. Although objective measurements—such as actigraphy or the use of wearable devices—could provide more accurate and reliable data, these methods were not feasible for our large-scale cohort. Second, the analysis may be subject to residual confounding, as not all potential covariates were considered. Factors such as physical activity, sleep medication, circadian rhythm disruptions, and specific interventions were not fully accounted for, due to high rates of missing data or lack of availability, potentially influencing the observed association between sleep duration and OA. Additionally, this is a cross-sectional study, therefore failed to provide a robust causal inference.

## Conclusion

Short sleep was associated with a higher incidence of OA and a U-shaped association was observed between sleep duration and OA incidence in middle-aged and older Chinese adults. BMI may act as a moderator in this relationship.

## Abbreviations

BMI: body mass index
CHARLS: China Health and Retirement Longitudinal Study
CI: confidence interval
CRP: C-reactive protein
DM: diabetes mellitus
IL-6: interleukin-6
OA: osteoarthritis
OR: odds ratio
SD: standard deviation
TNF-α: tumor necrosis factor-alpha
WBC: white blood cell count
WHO: World Health Organization
